# Robot-associated inpatient hospital activity in Germany, 2019–2025: nationwide trends in coded robot categories and diagnostic case mix

**DOI:** 10.1007/s11701-026-04084-z

**Published:** 2026-10-20

**Authors:** Roland Mojica Crespo

**Affiliations:** 1 Vascular Surgery, Faculty of Medicine, University of Augsburg, Augsburg, Germany; 2 Department of Vascular and Endovascular Surgery, University Hospital Augsburg, Stenglinstr. 2 86156 Augsburg, Germany

**Keywords:** Robotic surgery, Robot-assisted surgery, Germany, Nationwide analysis, Hospital data, Surgical trends

## Abstract

Robot-assisted surgery has spread from urology into several other surgical fields, and different categories of robotic system are now coded separately in Germany. This nationwide descriptive time-series analysis used aggregated hospital data of the Institute for the Hospital Remuneration System for 2019 to 2025. All inpatient hospital cases carrying at least one code of the group for use of a surgical robot (group 5-987 of the German Operation and Procedure Classification) were included, and annual case volume, the coded robot categories, principal diagnoses grouped at the three-character level, sex, age, clinical complexity and length of stay were described, together with selected index procedures counted with and without a robot code. The annual number of cases increased 3.78-fold, from 32,691 in 2019 to 123,706 in 2025, and rose in every year. Prostate cancer remained the most frequent principal diagnosis group: its case number almost doubled from 14,796 to 29,407 while its share fell from 45.3% to 23.8%. Osteoarthritis of knee became the second most frequent, rising from 1,936 to 21,447 cases. The proportion of female cases increased from 26.1% to 40.9%. Among hospital cases with the code for radical prostatovesiculectomy, the proportion also carrying a robot code rose from 52.9% to 85.1%, and for knee endoprosthesis implantation from 1.1% to 8.9%. Recorded robot-associated inpatient hospital activity almost quadrupled over seven years, and both the coded robot categories and the diagnostic composition broadened. These data describe recorded activity and its composition but do not provide information on outcomes, costs or appropriateness.

## Introduction

Robot-assisted surgery was adopted first and most widely in urology, where it became the standard approach to radical prostatectomy in several health systems [1]. Its use has since extended to general, colorectal, gynaecological, thoracic and orthopaedic operations. In the United States, national robotic case volume doubled between 2008 and 2013 [2], and in a statewide registry the robotic share of common general surgical procedures rose from 1.8% to 15.1% between 2012 and 2018 [3]. National series from Taiwan, France and the Netherlands describe rapid growth and a changing distribution across specialties, with urology the largest single field where specialty shares were reported [4–6]. Case counts alone do not show which kinds of robotic system are used, or for which conditions, and specialty labels are too broad to answer either question.

Where procedure coding identifies robotic assistance, national administrative data can measure this development across a whole health system [4, 5], and German coding is unusually informative, because the procedure group for use of a surgical robot separates complex console systems, robotic arms, miniature robots fixed to bone and a residual category [7]. For Germany, the evidence is nevertheless fragmented. A national survey identified 121 hospitals offering robot-assisted surgery at the end of 2018, most in urology [8]. The number of robotic systems in German hospitals roughly doubled between 2018 and 2022, but the StuDoQ|Robotics registry recorded only 3.7% to 36.7% of the robot-assisted visceral operations documented in national data based on diagnosis-related groups (DRG) [9]. Analyses of national hospital billing data have concentrated on individual specialties or patient groups, such as urological surgery [10], total knee arthroplasty [11] and children [12]. An all-specialty description of German hospital data covering 2005 to 2020 has been published as a conference abstract [13], but a peer-reviewed full-length analysis of the coded robot categories and the diagnostic case mix was not identified.

The aim of this study was to describe changes in robot-associated inpatient hospital activity in Germany between 2019 and 2025, including national case volume, the coded robot categories, the principal-diagnosis case mix, demographic and clinical characteristics, and, for selected index procedures, the proportion of hospital cases also carrying a robot code. National procedure-code statistics from 2005 onwards are reported separately as context.

## Methods

### Study design and data sources

This nationwide descriptive time-series analysis used two national data sources, kept separate throughout. The primary dataset is the DatenBrowser of the Institute for the Hospital Remuneration System (Institut für das Entgeltsystem im Krankenhaus, InEK). Under § 21 of the German Hospital Remuneration Act (Krankenhausentgeltgesetz), hospitals transmit case data for each preceding calendar year to a national data office run by InEK [14]. The DatenBrowser publishes aggregated results of these deliveries: for a selected group of cases it reports the case number, the distributions of sex, age class, clinical complexity level and duration of stay, and lists of principal diagnoses [15]. Each annual data set comprises the full and partial inpatient cases of the DRG remuneration sector discharged in that calendar year, with diagnoses coded in the International Classification of Diseases, 10th Revision, German Modification (ICD-10-GM) and procedures in the Operation and Procedure Classification (OPS), each in the version valid for that year. Data were analysed by data year, the calendar-year discharge period, for 2019 to 2025, the years for which the DatenBrowser provides these data. One query per data year used the robot procedure codes valid in that year as the only selection criterion, without clinical exclusions. The cohort data for 2019 to 2024 were retrieved on 10 August 2026 and reviewed on 17 September 2026, and those for 2025, together with the principal-diagnosis and index-procedure queries for all data years, on 7 and 8 October 2026. Throughout, robot-associated describes the hospital cases of this cohort, because the data record a documented robot code rather than the technique used, while robot-assisted refers to the clinical technique and to findings reported elsewhere.

The contextual dataset is the national DRG statistics of the Federal Statistical Office, which publish procedure codes at the four-digit OPS level for 2005 onwards [16, 17]. These statistics count each terminal code once per hospital case and add the terminal codes of a group, so a case documented with more than one robot subcode contributes more than once. They are therefore a national code frequency rather than a count of unique hospital cases; they are reported separately from the primary cohort, and the two are never combined into one series or one growth measure. Reporting was guided by the RECORD statement for studies using routinely collected health data [18].

### Study population and OPS robot categories

The primary cohort comprises all unique inpatient hospital cases carrying at least one valid OPS subcode of group 5-987 (application of a surgical robot) in the respective data year. In the OPS versions 2021 to 2024 the group contained four subcodes: 5-987.0 for a complex console-operated robot controlling articulated instruments with at least seven degrees of freedom and three or more endoscopic instruments with three-dimensional camera guidance; 5-987.1 for a robotic arm with at least six degrees of freedom; 5-987.2 for a miniature robot fixed to bone that prepares bone or joint surfaces automatically; and 5-987.x for other robot applications [7]. The definition of 5-987.1 was broadened with the OPS version 2021 from one mandatory feature to any one of three, and 5-987.2 was introduced with the same version, so years before 2021 are years without the code rather than without such procedures. In the OPS version 2025 the complex-robot category, whose criteria were unchanged from 2019 to 2024 and were not altered by the change, was subdivided by the number of arms into 5-987.00 and 5-987.01, and a code for a simple single-arm robot, 5-987.3, was introduced [7]; subcode counts are therefore reported separately before and after that version. The residual category 5-987.x carries no technical definition. Because a hospital case could carry more than one 5-987 subcode, subcode frequencies were not mutually exclusive and were not summed; each annual total is the unique case number reported by the combined query. The unit of analysis was the hospital case, counted once even if a code was documented more than once during the stay. The source contains no patient identifiers, so repeated admissions, staged procedures and transfers could not be linked.

### Variables and outcomes

The primary outcome was the annual number of unique hospital cases in the cohort. Secondary variables were the frequency of each robot subcode, the principal diagnosis, sex, age, Patient Clinical Complexity Level (PCCL) and length of stay (LOS). Sex was analysed as recorded; the categories diverse and not stated remained in the denominator but are not shown separately, because each comprised fewer than ten cases per year. The age classes of the source were combined into five groups: <18, 18–49, 50–64, 65–79 and ≥ 80 years. The PCCL is a case-level measure of the cumulative severity of comorbidities and complications on a scale from 0 to 6, assigned by the DRG grouping algorithm in the version of the respective data year [19], grouped here as 0, 1–2 and ≥ 3. The LOS was taken as the annual mean and standard deviation (SD) reported by the source; no median is available. Case numbers for sex, age and PCCL were derived from the published proportions; these permitted exact recovery of integer counts, which reconciled to the annual totals, so no imputation was required.

### Principal-diagnosis analysis

Principal diagnoses were analysed as three-character ICD-10-GM categories in the version valid for each data year [20], so that a condition recorded under several terminal codes appears once. Candidate categories were screened from the annual diagnosis lists and then counted by one targeted query per category and data year, so the reported values are exact counts rather than sums of listed rows. Because the screening used the individually listed diagnoses, a category whose cases lie mostly in suppressed rows could be ranked too low. The twenty highest-frequency categories among the screened candidates, ranked on pooled frequency across the seven data years rather than in a single year, were compared for 2019 and 2025 with their ICD-10-GM codes and English titles (Table 1); the ten most frequent were additionally displayed for all seven years. For each category, the annual number of cases, its share of annual cases and the change between 2019 and 2025 were described. Remaining cases were combined as all other principal diagnoses, a residual group and not a diagnosis. Outside the index-procedure queries, the source publishes separate marginal distributions, so a principal diagnosis cannot be linked to an individual robot subcode. The source lists diagnoses individually only if they include at least five cases in a year and reports the rest in a combined row [15]. These cases, 1.3% to 2.8% of annual cases, remained in the denominator, were counted within all other principal diagnoses and were not reconstructed.

**Table 1:** Twenty principal-diagnosis groups of robot-associated inpatient hospital cases with the highest pooled frequency among screened candidates, 2019 and 2025

ICD-10-GM group	Principal diagnosis	2019, *n* (%)	2025, *n* (%)	Absolute change, *n*
C61	Malignant neoplasm of prostate	14,796 (45.3)	29,407 (23.8)	+ 14,611
M17	Osteoarthritis of knee	1,936 (5.9)	21,447 (17.3)	+ 19,511
C64	Malignant neoplasm of kidney, except renal pelvis	2,596 (7.9)	8,064 (6.5)	+ 5,468
C18	Malignant neoplasm of colon	538 (1.6)	4,906 (4.0)	+ 4,368
C20	Malignant neoplasm of rectum	843 (2.6)	3,840 (3.1)	+ 2,997
N13	Obstructive and reflux uropathy	1,231 (3.8)	2,839 (2.3)	+ 1,608
K57	Diverticular disease of intestine	479 (1.5)	3,190 (2.6)	+ 2,711
K44	Diaphragmatic hernia	371 (1.1)	3,261 (2.6)	+ 2,890
D25	Leiomyoma of uterus	569 (1.7)	2,838 (2.3)	+ 2,269
N81	Female genital prolapse	487 (1.5)	2,710 (2.2)	+ 2,223
C67	Malignant neoplasm of bladder	521 (1.6)	2,235 (1.8)	+ 1,714
D30	Benign neoplasm of urinary organs	580 (1.8)	1,961 (1.6)	+ 1,381
C54	Malignant neoplasm of corpus uteri	398 (1.2)	2,206 (1.8)	+ 1,808
N40	Hyperplasia of prostate	555 (1.7)	1,147 (0.9)	+ 592
C16	Malignant neoplasm of stomach	231 (0.7)	2,046 (1.7)	+ 1,815
E66	Obesity	167 (0.5)	2,113 (1.7)	+ 1,946
C34	Malignant neoplasm of bronchus and lung	268 (0.8)	2,112 (1.7)	+ 1,844
K80	Cholelithiasis	222 (0.7)	593 (0.5)	+ 371
C15	Malignant neoplasm of oesophagus	308 (0.9)	1,439 (1.2)	+ 1,131
K43	Ventral hernia	98 (0.3)	1,502 (1.2)	+ 1,404
	All other principal diagnoses^a^	5,497 (16.8)	23,850 (19.3)	+ 18,353

Groups are three-character ICD-10-GM categories, ordered by pooled frequency across 2019–2025 among the candidates screened as described in the Methods. Percentages refer to all robot-associated inpatient hospital cases in the respective year (2019, *n* = 32,691; 2025, *n* = 123,706) and were calculated from the underlying counts, so they may not sum exactly. ^a^ All principal diagnoses other than the twenty listed, including diagnoses with fewer than five cases per year that the data source does not list individually. ICD-10-GM International Classification of Diseases, 10th Revision, German Modification. Source: InEK DatenBrowser

### Index procedures

The source was queried for hospital cases carrying both a principal diagnosis category and an index procedure code: prostate cancer (C61) with radical prostatovesiculectomy (5-604), kidney cancer (C64) with partial kidney resection (5-553) and with nephrectomy (5-554), and osteoarthritis of knee (M17) with knee endoprosthesis implantation (5-822). Each query was repeated with any valid 5-987 subcode of that year added, and the two index procedures were also queried without a diagnosis restriction, with and without the robot criterion, giving a denominator from the same source and unit. The resulting proportions count codes documented during the same hospital stay; they describe coded activity and do not establish that the robot code applied to the index procedure.

### Statistical analysis

The analysis was descriptive. Results are reported as counts and percentages of annual cases, with absolute and relative changes between 2019 and 2025, and the overall increase also as a fold change and a compound annual growth rate. Given the descriptive objective and the complete enumeration of recorded cases, no hypothesis tests, confidence intervals or trend models were used. Percentages and LOS values were rounded to one decimal place. Analyses were performed in Python 3.14.6 with the openpyxl (3.1.5) and matplotlib (3.11.1) libraries.

### Ethics

The study used exclusively aggregated, non-identifiable hospital case data. No individual-level data were accessed, and ethics approval and individual consent were not applicable.

## Results

### National case volume

Between 2019 and 2025, 487,689 unique inpatient hospital cases carried at least one OPS 5-987 subcode. The annual number increased from 32,691 in 2019 to 37,834 in 2020, 48,727 in 2021, 60,605 in 2022, 81,963 in 2023, 102,163 in 2024 and 123,706 in 2025 (Table 2). This corresponds to an increase of 91,015 cases (278.4%); the annual number increased 3.78-fold, at a compound annual growth rate of 24.8%, and rose in every year, by the smallest margin in 2020 (15.7%) and the largest in 2023 (35.2%).

**Table 2:** Annual characteristics of robot-associated inpatient hospital cases, 2019–2025

Characteristic	2019	2020	2021	2022	2023	2024	2025
Hospital cases, n	32,691	37,834	48,727	60,605	81,963	102,163	123,706
OPS 5-987 subcodes, n (%)^a^							
5-987.0 Complex surgical robot	28,815 (88.1)	33,059 (87.4)	41,655 (85.5)	51,048 (84.2)	69,078 (84.3)	85,827 (84.0)	–
5-987.00 Complex, single-arm	–	–	–	–	–	–	5,914 (4.8)
5-987.01 Complex, multi-arm	–	–	–	–	–	–	95,347 (77.1)
5-987.1 Robotic arm	3,042 (9.3)	3,746 (9.9)	5,627 (11.5)	7,430 (12.3)	10,110 (12.3)	12,379 (12.1)	16,896 (13.7)
5-987.2 Miniature robot	–	–	120 (0.2)	545 (0.9)	802 (1.0)	1,550 (1.5)	2,001 (1.6)
5-987.3 Simple single-arm robot	–	–	–	–	–	–	511 (0.4)
5-987.x Other	896 (2.7)	1,071 (2.8)	1,392 (2.9)	1,655 (2.7)	2,083 (2.5)	2,492 (2.4)	3,188 (2.6)
Sex, n (%)^b^							
Male	24,167 (73.9)	27,380 (72.4)	33,686 (69.1)	40,667 (67.1)	52,479 (64.0)	62,858 (61.5)	73,127 (59.1)
Female	8,522 (26.1)	10,453 (27.6)	15,041 (30.9)	19,938 (32.9)	29,479 (36.0)	39,298 (38.5)	50,575 (40.9)
Age group, n (%)							
< 18 years	132 (0.4)	130 (0.3)	159 (0.3)	194 (0.3)	262 (0.3)	418 (0.4)	489 (0.4)
18–49 years	3,652 (11.2)	4,003 (10.6)	5,552 (11.4)	6,978 (11.5)	9,678 (11.8)	12,577 (12.3)	14,987 (12.1)
50–64 years	12,113 (37.1)	13,924 (36.8)	17,613 (36.1)	21,163 (34.9)	28,352 (34.6)	34,360 (33.6)	40,852 (33.0)
65–79 years	15,277 (46.7)	17,652 (46.7)	22,115 (45.4)	27,751 (45.8)	37,263 (45.5)	46,490 (45.5)	57,164 (46.2)
≥ 80 years	1,517 (4.6)	2,125 (5.6)	3,288 (6.7)	4,519 (7.5)	6,408 (7.8)	8,318 (8.1)	10,214 (8.3)
PCCL, n (%)							
0	21,588 (66.0)	24,947 (65.9)	33,285 (68.3)	42,331 (69.8)	59,297 (72.3)	74,231 (72.7)	91,254 (73.8)
1–2	6,924 (21.2)	8,202 (21.7)	9,525 (19.5)	11,680 (19.3)	14,831 (18.1)	18,090 (17.7)	20,560 (16.6)
≥ 3	4,179 (12.8)	4,685 (12.4)	5,917 (12.1)	6,594 (10.9)	7,835 (9.6)	9,842 (9.6)	11,892 (9.6)
Length of stay, days							
Mean	8.4	8.1	7.9	7.7	7.6	7.6	7.5
SD	7.6	7.8	7.8	7.8	8.0	7.9	7.9

^a^A hospital case can carry more than one OPS 5-987 subcode, so subcode frequencies are not mutually exclusive, do not sum to the annual case number and were not used to derive it. 5-987.2 did not exist before the OPS version 2021. In the OPS version 2025 the complex-robot code 5-987.0 was subdivided into 5-987.00 and 5-987.01 and the code 5-987.3 was introduced, so the subcode series are not continuous across that version. A dash marks a code that did not exist in that year. ^b^ Cases recorded as diverse or with sex not stated were fewer than ten in every year and are not shown. OPS Operation and Procedure Classification; PCCL Patient Clinical Complexity Level; SD standard deviation. Source: InEK DatenBrowser

**Fig. 1:**
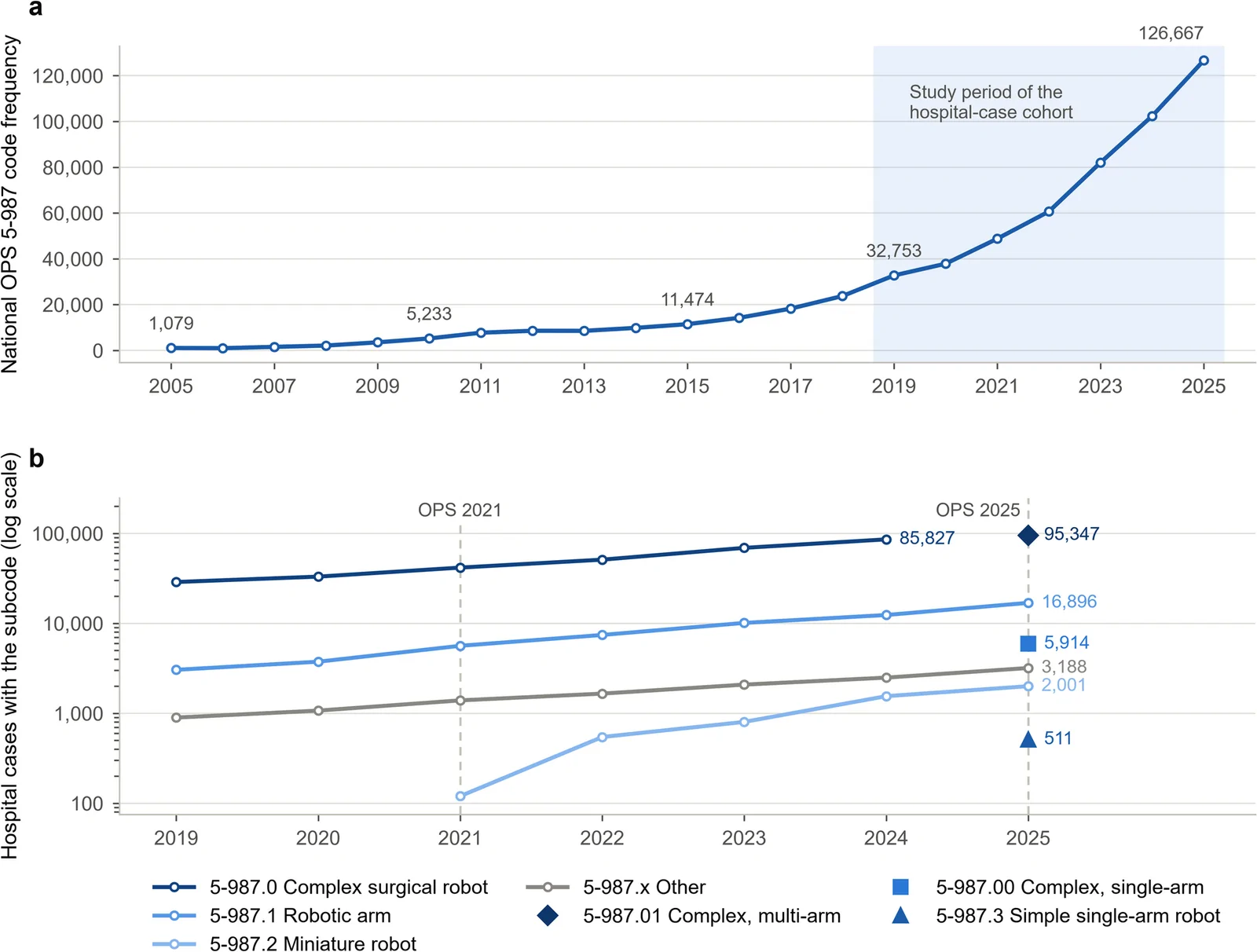
Robot-associated procedure coding in Germany. (**a**) National OPS 5-987 code frequency, 2005–2025, from the national DRG statistics; these statistics count terminal procedure codes once per hospital case and add them within the code group, so they are a code frequency and not a count of unique hospital cases, and the shaded band marks the period covered by the hospital-case cohort of this study. (**b**) Annual number of unique hospital cases carrying each OPS 5-987 subcode, 2019–2025, on a logarithmic scale; a case can carry more than one subcode, so the series are not mutually exclusive and do not sum to the annual cohort. The dashed lines mark the OPS versions 2021 and 2025. In the OPS version 2021 the definition of 5-987.1 was broadened and 5-987.2 was introduced, so 5-987.2 is plotted from 2021 onwards rather than as zero. In the OPS version 2025 the complex-robot code 5-987.0 was subdivided into 5-987.00 and 5-987.01 and the code 5-987.3 was introduced, so the series of 5-987.0 ends in 2024 and the three codes of 2025 are shown separately and are not joined to it. OPS Operation and Procedure Classification. Sources: (a) Statistisches Bundesamt; (b) InEK DatenBrowser

The national OPS 5-987 code frequency, which counts documented codes rather than unique cases and is reported here only as context, rose from 1,079 in 2005 to 126,667 in 2025 [16, 17] (Fig. 1a). The long-term increase was marked but not uninterrupted: the national count fell slightly in 2006 and again in 2013.

### OPS robot categories

Cases coded with a complex robot (5-987.0) rose from 28,815 in 2019 to 85,827 in 2024, the largest category in each of those years, falling from 88.1% to 84.0% of annual cases (Fig. 1b; Table 2). This code was subdivided in the OPS version 2025, so its series ends with the data year 2024. In 2025 the multi-arm code 5-987.01 was documented in 95,347 cases (77.1%), the most frequent subcode of that year, and the single-arm code 5-987.00 in 5,914 (4.8%). Cases coded with a robotic arm (5-987.1) rose from 3,042 to 16,896, or from 9.3% to 13.7% of annual cases, with a marked increase between 2020 and 2021, when the definition of this subcode was broadened. The miniature-robot code (5-987.2) existed from 2021, rising from 120 to 2,001 cases (1.6%), and the code for a simple single-arm robot, introduced in 2025, was documented in 511 cases (0.4%). The residual category 5-987.x rose from 896 to 3,188 cases, 2.6% of cases in 2025. Because a case can carry more than one subcode, these shares do not sum to 100%: the subcode sum exceeded the number of unique cases by 42 to 151 cases per year, or 0.08% to 0.19%.

### Principal-diagnosis case mix

**Fig. 2:**
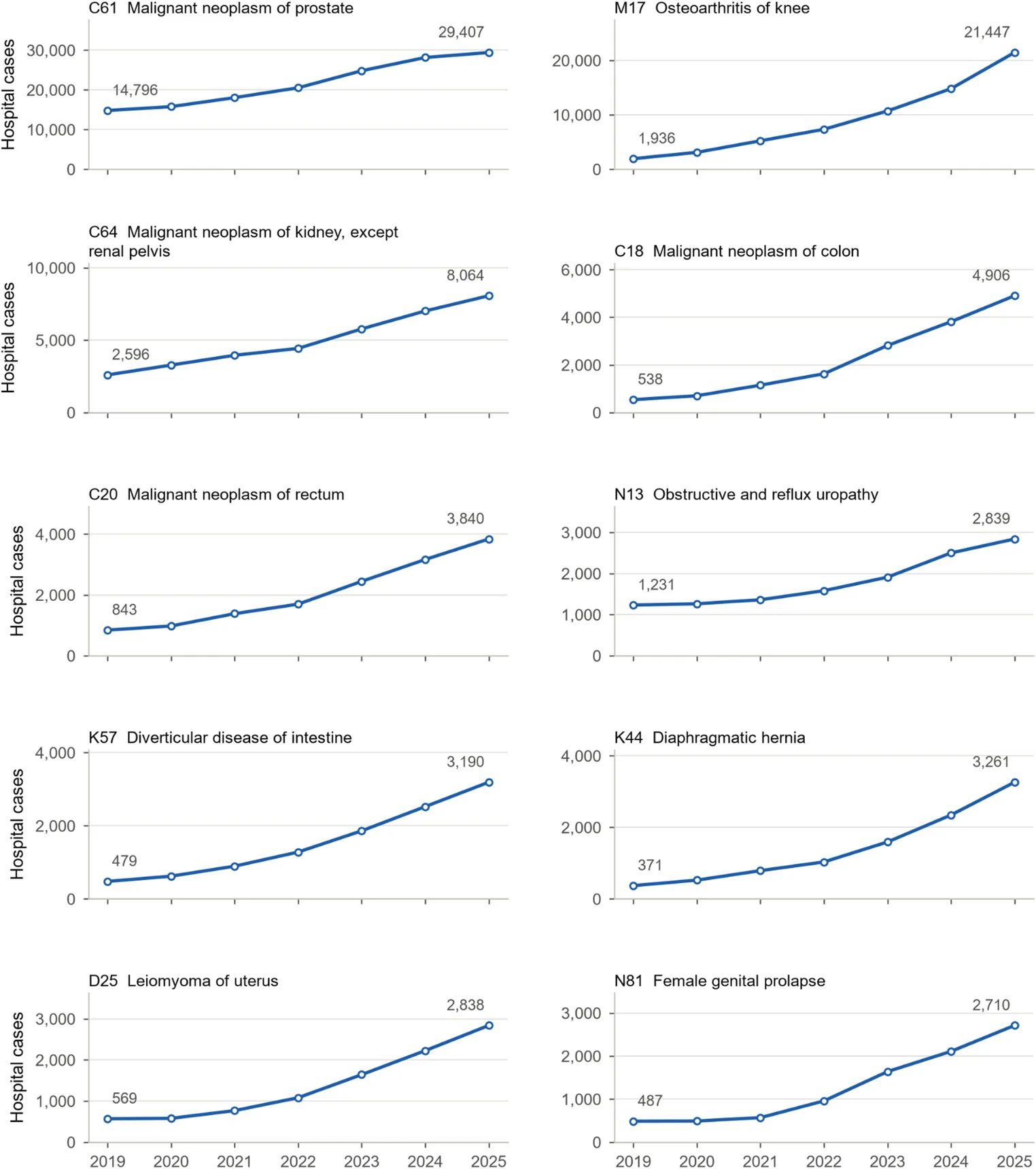
Ten principal-diagnosis groups of robot-associated inpatient hospital cases in Germany with the highest pooled frequency among screened candidates, 2019–2025. Annual number of cases for the ten three-character ICD-10-GM categories with the highest pooled frequency among the candidates screened as described in the Methods; the y-axes are scaled separately for each panel, and labels show the 2019 and 2025 values. The figure shows the ten leading groups for all seven years, whereas Table 1 compares 2019 and 2025 for the twenty leading groups. ICD-10-GM International Classification of Diseases, 10th Revision, German Modification. Source: InEK DatenBrowser

Malignant neoplasm of prostate (C61) was the most frequent principal diagnosis group in every year (Fig. 2; Table 1). The number of cases in this group almost doubled from 14,796 in 2019 to 29,407 in 2025, an increase of 14,611 cases, whereas its share of all cases fell from 45.3% to 23.8% and declined in every year. Osteoarthritis of knee (M17) ranked second by pooled frequency across the seven data years, rising from 1,936 cases (5.9%) in 2019 to 21,447 (17.3%) in 2025. Malignant neoplasm of kidney, except renal pelvis (C64) ranked third on the same pooled basis, with case numbers rising from 2,596 to 8,064 while its share fell from 7.9% to 6.5%.

Table 1 compares 2019 and 2025 for the twenty highest-frequency groups among the screened candidates. The ten leading groups accounted for 72.9% of cases in 2019 and 66.7% in 2025, the groups ranked eleventh to twentieth for 10.2% and 14.0%, and the twenty together for 83.2% and 80.7%. Among the twenty, the largest gains in share were those of osteoarthritis of knee, of malignant neoplasm of colon (C18, from 1.6% to 4.0%) and of diaphragmatic hernia (K44, from 1.1% to 2.6%).

### Index procedures

Among hospital cases carrying the code for radical prostatovesiculectomy (5-604), the proportion also carrying a 5-987 code rose from 52.9% in 2019 to 85.1% in 2025, and for knee endoprosthesis implantation (5-822) from 1.1% to 8.9% (Table 3). Restricted to cases with the corresponding principal diagnosis, the proportions rose from 53.2% to 85.3% and from 1.1% to 9.1%. For kidney cancer, the proportion was higher with partial kidney resection (29.0% to 68.3%) than with nephrectomy (6.9% to 36.4%) in every year.

**Table 3:** Hospital cases carrying an index procedure code and an OPS 5-987 code, 2019–2025

Analysis	2019	2020	2021	2022	2023	2024	2025
Principal diagnosis and index procedure							
Malignant neoplasm of prostate (C61) with radical prostatovesiculectomy (OPS 5-604)	14,467/27,178 (53.2)	15,419/26,551 (58.1)	17,663/27,432 (64.4)	20,205/29,363 (68.8)	24,514/32,303 (75.9)	27,845/34,355 (81.1)	29,152/34,171 (85.3)
Malignant neoplasm of kidney (C64) with partial kidney resection (OPS 5-553)	2,070/7,150 (29.0)	2,651/7,130 (37.2)	3,092/7,254 (42.6)	3,448/7,212 (47.8)	4,455/8,272 (53.9)	5,346/8,582 (62.3)	6,022/8,811 (68.3)
Malignant neoplasm of kidney (C64) with nephrectomy (OPS 5-554)	418/6,034 (6.9)	559/5,568 (10.0)	773/5,466 (14.1)	899/5,254 (17.1)	1,212/5,287 (22.9)	1,531/5,232 (29.3)	1,913/5,254 (36.4)
Osteoarthritis of knee (M17) with knee endoprosthesis implantation (OPS 5-822)	1,932/169,356 (1.1)	3,096/151,115 (2.0)	5,225/149,941 (3.5)	7,321/176,146 (4.2)	10,696/203,599 (5.3)	14,774/220,185 (6.7)	21,406/234,596 (9.1)
Index procedure, all principal diagnoses							
Radical prostatovesiculectomy (OPS 5-604), all principal diagnoses	14,524/27,454 (52.9)	15,492/26,784 (57.8)	17,722/27,633 (64.1)	20,276/29,576 (68.6)	24,590/32,528 (75.6)	27,950/34,679 (80.6)	29,271/34,395 (85.1)
Knee endoprosthesis implantation (OPS 5-822), all principal diagnoses	1,948/177,342 (1.1)	3,120/158,395 (2.0)	5,252/156,912 (3.3)	7,354/183,091 (4.0)	10,731/211,355 (5.1)	14,908/228,278 (6.5)	21,520/243,067 (8.9)

Each cell gives the number of hospital cases in which both the index procedure code and any OPS 5-987 code were documented during the same stay, over the number of hospital cases carrying the index procedure code, with the resulting percentage. Documentation of both codes in the same hospital case does not establish that the robot code applied to the index procedure, so these proportions describe coded activity and are not measures of how often these operations were performed robotically. Numerator and denominator come from the same source and the same unit, the hospital case. OPS Operation and Procedure Classification; C61 malignant neoplasm of prostate; C64 malignant neoplasm of kidney, except renal pelvis; M17 osteoarthritis of knee. Source: InEK DatenBrowser

### Demographic characteristics

**Fig. 3:**
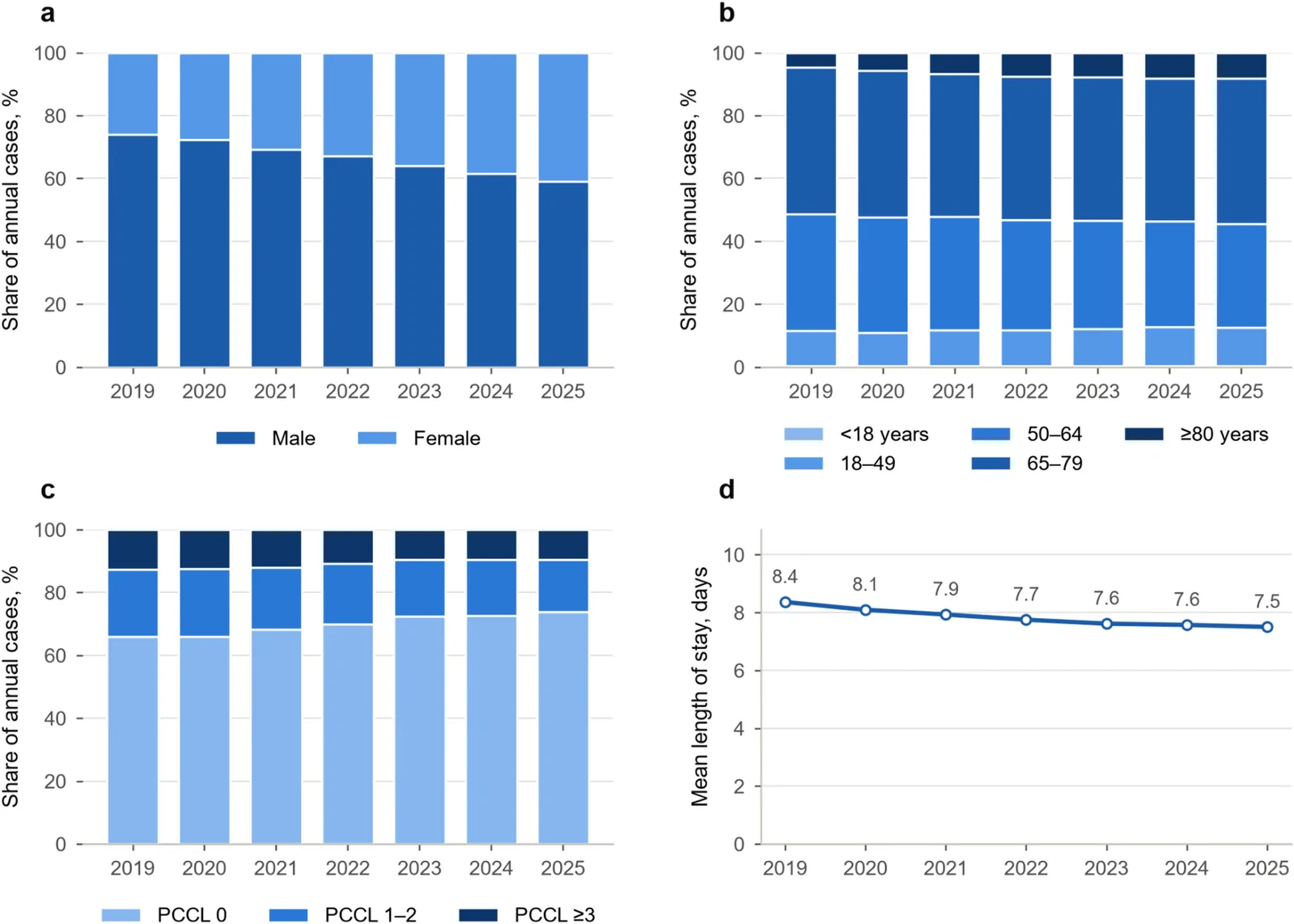
Demographic and clinical characteristics of robot-associated inpatient hospital cases in Germany, 2019–2025. (**a**) Share of male and female cases; cases recorded as diverse or with sex not stated are not shown. (**b**) Share of cases by age group. (**c**) Share of cases by Patient Clinical Complexity Level (PCCL) group. (**d**) Annual mean length of stay as reported by the source; the standard deviation is given in Table 2. Percentages refer to all cases in the respective year. Source: InEK DatenBrowser

The proportion of female cases increased from 26.1% in 2019 to 40.9% in 2025, and the proportion of male cases decreased accordingly (Fig. 3a; Table 2). Both groups grew in absolute number: male cases from 24,167 to 73,127 and female cases from 8,522 to 50,575. The share of cases aged ≥ 80 years increased from 4.6% to 8.3% (1,517 to 10,214 cases) and the share aged 18–49 years from 11.2% to 12.1%, while the share aged 50–64 years decreased from 37.1% to 33.0% and the share aged 65–79 years from 46.7% to 46.2% (Fig. 3b). The 65–79 group remained the largest in every year, and all five age groups increased in absolute number.

### Clinical complexity and length of stay

The share of cases with a PCCL of 0 increased from 66.0% to 73.8%, whereas the shares with a PCCL of 1–2 and ≥ 3 decreased from 21.2% to 16.6% and from 12.8% to 9.6% (Fig. 3c; Table 2). The number of cases with a PCCL of ≥ 3 nevertheless rose from 4,179 to 11,892. The mean LOS decreased from 8.4 days in 2019 to 7.5 days in 2025, a change of − 0.9 days (Fig. 3d). The LOS SD ranged from 7.6 to 8.0 days.

## Discussion

Between 2019 and 2025, the number of inpatient hospital cases in Germany carrying a code for use of a surgical robot almost quadrupled and increased in every year. The coded robot categories broadened: complex robots remained the large majority of cases, the robotic-arm category increased in frequency and share, a miniature-robot class appeared in 2021 and the complex-robot code was subdivided in 2025. Prostate cancer remained the leading principal diagnosis group and its case number almost doubled, but its share of all cases fell by almost half, and osteoarthritis of knee became the second most frequent group. Among hospital cases carrying selected index procedure codes, the proportion also carrying a robot code rose in every analysis. Over the same period the recorded cases included more women and more cases aged 80 years or older, a lower proportion with high clinical complexity, and a slightly shorter mean stay. These findings describe recorded activity and case composition and do not explain the reasons for the changes.

The direction of this growth corresponds to that reported from other health systems, where recorded activity increased 23-fold in Taiwan between 2011 and 2024 [4] and approximately tenfold in the United Kingdom between 2014 and 2024 [21], with national series from the United States, France and the Netherlands pointing the same way [2, 5, 6]. These studies differ from the present analysis in coding systems, units of analysis, denominators and observation periods, so their magnitudes are not directly comparable. The national code statistics place the present cohort within a much longer German development, from 1,079 codes in 2005 to 126,667 in 2025. An earlier all-specialty description reported the same national development up to 2020 as a conference abstract [13]; the present analysis extends it to 2025 and adds a deduplicated hospital-case cohort, the separation of the coded robot categories, grouped principal diagnoses and index-procedure denominators. The smallest annual increase occurred in 2020, the first year of the COVID-19 pandemic, when most responding German departments reported fewer robotic visceral operations than in 2019 in a survey with 17 analysable responses [22]. The aggregate data do not allow this pattern to be attributed to the pandemic.

The broadening of the coded robot categories is the feature the earlier single-code view could not show, and it must be read with the classification in mind. The complex-robot criteria were not altered by the subdivision in 2025, but a separate category for simple single-arm robots was introduced in the same version, so counts before and after it are reported separately. The robotic-arm class is not interpretable throughout: its definition was broadened in 2021, so systems offering only automatic positioning or tracking became codable without a change in practice, and part of the 2020 to 2021 step may be classificatory. The miniature-robot class has no pre-2021 baseline, so its appearance must not be read as the introduction of the technology into practice, and the residual category collects everything without a specific definition. Within these limits, the non-console categories represent a measurable and growing component of recorded robot-associated activity.

The change in diagnostic composition reflects growth outside prostate cancer rather than a decline in prostate cancer cases, which almost doubled. This is consistent with the early and sustained role of robotic radical prostatectomy [1, 23] and with the continued growth of robot-assisted urological surgery in German national data [10]. The increasing contribution of other diagnoses mirrors the spread of robotic techniques into general, colorectal, gynaecological and foregut surgery in other countries [3, 6, 24–26]. German data point in the same direction for endometrial cancer surgery, upper urinary tract reconstruction, diverticular disease and colorectal resections [9, 27–29], and robot-assisted approaches have also been described in kidney transplantation and living donor nephrectomy [30, 31]. The increased representation of osteoarthritis of knee coincided with the expansion of robot-code categories beyond the complex-robot code, although the aggregated data do not link diagnoses to specific robotic systems. A separate German analysis of OPS billing data has likewise reported a steep increase in robot-assisted total knee arthroplasty from a low base [11]. Six of the ten leading groups were non-malignant, so hospital cases with a documented surgical robot were not confined to malignant principal diagnoses.

The index-procedure analyses point the same way within defined procedure groups: by 2025 most hospital cases carrying the code for radical prostatovesiculectomy also carried a robot code, whereas for knee endoprosthesis implantation the proportion remained below one in ten, and for kidney cancer it was consistently higher with partial kidney resection than with nephrectomy.

The present data describe principal diagnoses, not operations, so changes in diagnostic composition do not by themselves show the adoption of specific operations. The rising proportion of female cases is compatible with the growth of gynaecological, orthopaedic and visceral diagnosis groups in this cohort, although the data cannot show that these changes account for it; the earlier German description reported a narrowing male-to-female ratio up to 2020 [13]. In an all-specialty cohort the increase in cases aged 80 years or older is likewise more plausibly read as a change in case mix than as rising age at a single operation, although increasing age at radical prostatectomy has been described in Germany and in Japan [32, 33]. The lower share of cases with a PCCL of 3 or higher and the shorter mean LOS should also be read descriptively: the national mean stay in Germany has declined slightly in recent years [34], and secular change, case mix and differences between robot categories cannot be separated with these data. Neither indicates that robotic surgery improved outcomes.

A national description of this kind complements registries that capture only part of robotic activity [9]. Annual case numbers, the coded robot categories and the diagnostic composition can support the monitoring of recorded robotic activity, the planning of operating theatre and robotic system capacity, and the planning of training, for which national curricula have been developed in Germany [35, 36]. The growing proportion of cases outside prostate cancer, together with the increasing frequency of non-console robot-code categories, means that such planning increasingly has to account for a wider range of clinical applications and coded robot categories sharing operating time and trained staff. Because the costs of new surgical technologies are often not fully covered within the German DRG system [37], information on the scale and distribution of robotic activity is also relevant to investment and resource allocation. Specialty-specific research linking robotic use with the operation performed and with patient outcomes will be needed to assess effectiveness and resource use, which descriptive case counts cannot address.

This study has limitations. It relies on administrative coding; the completeness and accuracy of the 5-987 documentation have not been validated against clinical records, and temporal changes in coding completeness may have influenced the magnitude of the observed trends. Four classification changes affect the coded robot categories, and the residual category is undefined by construction. Subcode frequencies are not mutually exclusive, and the overlap combinations cannot be reconstructed. Case numbers do not represent individual patients. Denominators are available only for the index procedures queried here, and those proportions count codes documented in the same hospital case rather than the technique used; outside those queries, diagnoses and procedures are published as separate distributions. Diagnoses with fewer than five cases per year are suppressed by the source, and because candidate categories were screened from the individually listed diagnoses, categories composed predominantly of suppressed rows could have been under-ranked. Clinical detail such as disease stage, operative time, complications and outcomes is unavailable. The data cover inpatient DRG-sector cases, not outpatient procedures, and the national code statistics used as context count codes rather than cases. Finally, the design is descriptive and does not support causal inference about the drivers or consequences of these changes.

The strengths are the national coverage of recorded DRG-sector hospital cases meeting the coding criterion, seven consecutive years of observation with 487,689 cases, a cohort defined by the full valid code group, the separate description of the coded robot categories, the grouping of principal diagnoses into clinical categories, and index-procedure denominators from the same source and unit. The extraction and analysis are reproducible from the aggregated sources named in the data availability statement.

## Conclusions

Recorded robot-associated inpatient hospital activity in Germany almost quadrupled between 2019 and 2025. The coded robot categories diversified beyond complex robots and the diagnostic case mix broadened: prostate cancer remained the leading group while its share fell substantially, and osteoarthritis of knee became the second most frequent. Among hospital cases carrying selected index procedure codes, the proportion also carrying a robot code increased. National administrative hospital data give a reproducible view of the scale and composition of robot-associated activity and can support monitoring and planning. They do not provide information on the effectiveness, safety or appropriateness of robot-assisted procedures, which require clinical and outcome data.

## Data Availability

The aggregated data analysed in this study are available through the InEK DatenBrowser of the Institute for the Hospital Remuneration System (Institut für das Entgeltsystem im Krankenhaus, InEK) and through the national DRG statistics of the Federal Statistical Office. The data were retrieved for data years 2019 to 2025 by selecting the OPS 5-987 subcodes valid in each year without further selection criteria, and, for the index procedures, by combining the diagnosis, procedure and robot codes valid in each year. Derived data underlying the reported tables and figures are available from the corresponding author upon reasonable request.

## Abbreviations

DRG: Diagnosis-related groups
ICD-10-GM: International Classification of Diseases, 10th Revision, German Modification
InEK: Institute for the Hospital Remuneration System
LOS: Length of stay
OPS: Operation and Procedure Classification
PCCL: Patient Clinical Complexity Level
SD: Standard deviation

## Code Availability

The analysis code is available from the corresponding author upon reasonable request.
